# Analysis of Gestational Weight Gain During the COVID-19 Pandemic in the US

**DOI:** 10.1001/jamanetworkopen.2022.30954

**Published:** 2022-09-09

**Authors:** Wangnan Cao, Shengzhi Sun, Valery A. Danilack

**Affiliations:** 1 Department of Social Medicine and Health Education, School of Public Health, Peking University, Beijing, China; 2 Department of Epidemiology and Biostatistics, School of Public Health, Capital Medical University, Beijing, China; 3 Department of Internal Medicine, Yale School of Medicine, New Haven, Connecticut

## Abstract

This cross-sectional study assesses changes in gestational weight gain in individuals giving birth to live infants during the COVID-19 pandemic.

## Introduction

The COVID-19 pandemic has been associated with weight gain among adults, children, and adolescents,^1,2^ but little is known about gestational weight gain (GWG) among pregnant individuals. Gestational weight gain is associated with important health implications for parents and offspring, and excessive GWG is associated with adverse pregnancy outcomes.^3^ We estimated changes in GWG among individuals giving birth to live infants during the COVID-19 pandemic in the US.

## Methods

In this cross-sectional study, we obtained data on all live births in the US from January 1, 2018, to December 31, 2020, from the National Center for Health Statistics of the Centers for Disease Control and Prevention. Data analysis was performed from January 1, 2022, to July 15, 2022. We restricted our analyses to singleton births to residents and excluded births with missing gestational age, GWG, and body mass index (BMI; calculated as weight in kilograms divided by height in meters squared) before pregnancy. Gestational weight gain was calculated by subtracting weight before pregnancy from the delivery weight. To examine vulnerable racial and ethnic groups, race and ethnicity were categorized on the basis of US birth certificate questionnaires as Hispanic, non-Hispanic Asian or non-Hispanic Pacific Islander, non-Hispanic Black, non-Hispanic White, and other race or ethnicity (including non-Hispanic American Indian or Alaskan Native, non-Hispanic with more than 1 race, and unknown or undisclosed race or ethnicity). Patient consent was waived because the study involved analysis of deidentified publicly available data and was deemed non–human participant research by the institutional review board at the Capital Medical University. This study followed the STROBE reporting guideline.

We defined the COVID-19 pandemic period as March 1 to December 31, 2020. We defined dichotomous excessive GWG as weight gain above the BMI-specific Institute of Medicine recommendations.^4^ We used linear regression or logistic regression to compare the GWG (continuous outcome) or excessive GWG (dichotomous outcome) among patients whose infants were born during the pandemic period vs the analogous period in 2019 (ie, referent period) after excluding prepandemic trends in GWG by comparing GWG or excessive GWG during the referent period in 2019 vs 2018 (eAppendix in the Supplement). We adjusted for gestational age, maternal age, educational attainment, race and ethnicity, marital status, adequacy of prenatal care utilization index, BMI before pregnancy, and source of delivery payment. Analyses were performed in R software, version 3.6.1 (R Foundation for Statistical Computing). A 2-sided *P* < .05 was considered statistically significant.

## Results

Our analysis included 2 847 592 singleton births in 2020 (mean [SD] GWG, 13.31 [6.85] kg), 2 475 822 in 2019 (mean [SD] GWG, 13.28 [6.84] kg), and 2 847 592 in 2018 (mean [SD] GWG, 13.31 [6.85] kg) (Table 1). After adjusting for covariates and excluding prepandemic trends in GWG, we observed an increase of 0.06 kg (95% CI, 0.04-0.07 kg) in GWG, with pronounced increases among pregnant individuals younger than 25 years (net change, 0.22; 95% CI, 0.19-0.26), non-Hispanic Black individuals (net change, 0.12; 95% CI, 0.07-0.16), unmarried individuals (net change, 0.16; 95% CI, 0.13-0.19), individuals who had obesity before pregnancy (net change, 0.17; 95% CI, 0.14-0.21), and individuals who used Medicaid to pay for delivery (net change, 0.17; 95% CI, 0.15-0.20) (Table 1). The pandemic was also associated with an increased risk of excessive GWG (ratio of odds ratio, 1.01; 95% CI, 1.01-1.02) (Table 2). The susceptible populations to excessive GWG were the same as for continuous GWG.

**Table 1.** Changes in Gestational Weight Gain (GWG) Before and During the COVID-19 Pandemic by Maternal Characteristics

Maternal characteristic	Prepandemic year 2018		Prepandemic year 2019		Pandemic year 2020		Adjusted changes in GWG, mean (95% CI), kg^a^		Net change during pandemic, mean (95% CI), kg^b^
	No. (%)	GWG, mean (SD), kg	No. (%)	GWG, mean (SD), kg	No. (%)	GWG, mean (SD), kg	2019 vs 2018	2020 vs 2019	
Total	2 475 822 (100)	13.28 (6.85)	2 486 122 (100)	13.26 (6.84)	2 847 592 (100)	13.31 (6.85)	0.03 (0.02 to 0.05)	0.09 (0.08 to 0.10)	0.06 (0.04 to 0.07)
Age, y									
<25	597 905 (24.1)	13.60 (7.31)	589 056 (23.7)	13.59 (7.30)	652 564 (22.9)	13.81 (7.36)	0.04 (0.01 to 0.07)	0.26 (0.24 to 0.29)	0.22 (0.19 to 0.26)
25-29	722 167 (29.2)	13.24 (6.91)	718 612 (28.9)	13.24 (6.91)	808 139 (28.4)	13.28 (6.91)	0.06 (0.03 to 0.08)	0.09 (0.07 to 0.11)	0.03 (0 to 0.06)
30-34	713 137 (28.8)	13.29 (6.54)	722 169 (29.0)	13.26 (6.53)	844 536 (29.7)	13.23 (6.53)	0.01 (−0.01 to 0.03)	0.01 (−0.01 to 0.03)	0 (−0.03 to 0.03)
≥35	442 613 (17.9)	12.91 (6.59)	456 285 (18.4)	12.89 (6.56)	542 353 (19.0)	12.87 (6.56)	0.02 (0 to 0.05)	0 (−0.03 to 0.02)	−0.02 (−0.06 to 0.01)
Educational attainment									
High school or less	937 032 (37.8)	12.76 (7.31)	939 208 (37.8)	12.73 (7.30)	1 071 703 (37.7)	12.86 (7.34)	0.03 (0.01 to 0.05)	0.19 (0.17 to 0.21)	0.16 (0.13 to 0.19)
Some college	492 995 (19.9)	13.32 (7.23)	485 850 (19.5)	13.33 (7.24)	539 398 (18.9)	13.45 (7.25)	0.08 (0.05 to 0.10)	0.17 (0.15 to 0.20)	0.10 (0.06 to 0.14)
Associate’s degree	207 092 (8.4)	13.38 (6.84)	207 219 (8.3)	13.35 (6.84)	237 831 (8.4)	13.39 (6.86)	0.04 (0.00 to 0.08)	0.09 (0.05 to 0.13)	0.05 (−0.01 to 0.10)
Bachelor’s degree	505 845 (20.4)	13.84 (6.10)	511 813 (20.6)	13.82 (6.08)	600 393 (21.1)	13.75 (6.10)	0.01 (−0.01 to 0.04)	−0.04 (−0.07 to −0.02)	−0.06 (−0.09 to −0.02)
Master’s degree or higher	302 663 (12.2)	13.93 (5.76)	308 812 (12.4)	13.89 (5.75)	362 104 (12.7)	13.73 (5.77)	0.00 (−0.03 to 0.02)	−0.14 (−0.17 to −0.12)	−0.14 (−0.18 to −0.10)
Unknown	30 195 (1.2)	12.43 (6.53)	33 220 (1.3)	12.41 (6.52)	36 163 (1.3)	12.42 (6.63)	0.02 (−0.08 to 0.12)	0.14 (0.05 to 0.23)	0.12 (−0.02 to 0.25)
Race or ethnicity									
Hispanic	563 214 (22.7)	12.31 (6.61)	581 543 (23.4)	12.30 (6.55)	686 707 (24.1)	12.40 (6.58)	0.04 (0.02 to 0.06)	0.12 (0.10 to 0.14)	0.08 (0.05 to 0.11)
Non-Hispanic Black	359 741 (14.5)	12.62 (7.61)	363 839 (14.6)	12.63 (7.60)	408 663 (14.4)	12.75 (7.57)	0.05 (0.02 to 0.08)	0.17 (0.14 to 0.20)	0.12 (0.07 to 0.16)
Non-Hispanic White	1 299 100 (52.5)	13.98 (6.76)	1 284 053 (51.6)	13.96 (6.77)	1 459 631 (51.3)	13.98 (6.78)	0.02 (0.00 to 0.03)	0.05 (0.04 to 0.07)	0.04 (0.01 to 0.06)
Other^c^	96 865 (3.9)	13.46 (7.31)	98 515 (4.0)	13.47 (7.31)	111 782 (3.9)	13.64 (7.41)	0.07 (0.01 to 0.13)	0.22 (0.16 to 0.28)	0.15 (0.06 to 0.23)
Marital status									
Married	1 256 522 (50.8)	13.38 (6.48)	1 257 871 (50.6)	13.36 (6.46)	1 496 696 (52.6)	13.32 (6.43)	0.02 (0.01 to 0.04)	0.01 (−0.01 to 0.02)	−0.01 (−0.04 to 0.01)
Unmarried	858 511 (34.7)	13.39 (7.54)	874 752 (35.2)	13.35 (7.55)	1 019 932 (35.8)	13.46 (7.57)	0.03 (0.01 to 0.06)	0.20 (0.18 to 0.22)	0.16 (0.13 to 0.19)
Unknown	360 789 (14.6)	12.71 (6.37)	353 499 (14.2)	12.71 (6.26)	330 964 (11.6)	12.76 (6.32)	0.07 (0.04 to 0.09)	0.12 (0.09 to 0.14)	0.05 (0.01 to 0.09)
APNCU index^d^									
Inadequate	330 942 (13.4)	12.20 (7.35)	336 462 (13.5)	12.18 (7.33)	386 160 (13.6)	12.29 (7.31)	0.05 (0.02 to 0.09)	0.14 (0.11 to 0.17)	0.09 (0.04 to 0.14)
Intermediate	76 071 (3.1)	12.78 (6.94)	73 084 (2.9)	12.74 (6.92)	115 526 (4.1)	12.91 (6.80)	0.03 (−0.04 to 0.10)	0.15 (0.09 to 0.21)	0.13 (0.03 to 0.22)
Adequate	313 184 (12.6)	13.46 (6.67)	307 518 (12.4)	13.40 (6.66)	419 867 (14.7)	13.51 (6.62)	0.01 (−0.03 to 0.04)	0.12 (0.09 to 0.15)	0.12 (0.07 to 0.16)
Adequate plus	1 698 163 (68.6)	13.49 (6.74)	1 714 243 (69.0)	13.47 (6.73)	1 863 179 (65.4)	13.50 (6.77)	0.03 (0.02 to 0.05)	0.07 (0.06 to 0.08)	0.04 (0.02 to 0.06)
Unknown	57 462 (2.3)	13.23 (7.41)	54 815 (2.2)	13.26 (7.33)	62 860 (2.2)	13.22 (7.36)	0.11 (0.02 to 0.19)	0.00 (−0.08 to 0.08)	−0.11 (−0.23 to 0.01)
BMI before pregnancy									
Underweight (<18.5)	81 528 (3.3)	15.10 (6.16)	78 554 (3.2)	15.18 (6.18)	83 528 (2.9)	15.11 (6.28)	0.10 (0.04 to 0.16)	−0.07 (−0.13 to −0.01)	−0.17 (−0.26 to −0.09)
Normal weight (18.5-24.9)	1 051 356 (42.5)	14.72 (6.00)	1 030 093 (41.4)	14.71 (6.00)	1 157 553 (40.7)	14.71 (6.03)	0.01 (0.00 to 0.03)	0.01 (−0.01 to 0.02)	−0.01 (−0.03 to 0.02)
Overweight (25.0-29.9)	657 042 (26.5)	13.48 (6.91)	666 751 (26.8)	13.51 (6.86)	770 820 (27.1)	13.58 (6.86)	0.04 (0.02 to 0.07)	0.09 (0.07 to 0.11)	0.04 (0.01 to 0.08)
Obesity (≥30)	685 896 (27.7)	10.67 (7.33)	710 724 (28.6)	10.72 (7.29)	835 691 (29.3)	10.93 (7.30)	0.05 (0.02 to 0.07)	0.22 (0.20 to 0.24)	0.17 (0.14 to 0.21)
Payment source for delivery									
Medicaid	1 034 348 (41.8)	12.87 (7.38)	1 031 578 (41.5)	12.84 (7.36)	1 179 455 (41.4)	12.99 (7.38)	0.04 (0.02 to 0.06)	0.21 (0.19 to 0.23)	0.17 (0.15 to 0.20)
Private insurance	1 235 811 (49.9)	13.69 (6.40)	1 251 825 (50.4)	13.67 (6.40)	1 443 554 (50.7)	13.61 (6.41)	0.03 (0.02 to 0.05)	−0.02 (−0.04 to −0.01)	−0.05 (−0.08 to −0.03)
Self-pay	99 501 (4.0)	12.42 (6.31)	102 183 (4.1)	12.39 (6.33)	108 310 (3.8)	12.52 (6.33)	0.03 (−0.02 to 0.08)	0.13 (0.08 to 0.19)	0.11 (0.03 to 0.18)
Other	95 451 (3.9)	13.40 (6.76)	88 249 (3.5)	13.42 (6.75)	97 904 (3.4)	13.58 (6.82)	0.01 (−0.05 to 0.07)	0.19 (0.13 to 0.24)	0.18 (0.09 to 0.26)
Unknown	10 711 (0.4)	13.50 (7.28)	12 287 (0.5)	13.45 (7.00)	18 369 (0.6)	13.47 (7.08)	−0.04 (−0.21 to 0.14)	0.09 (−0.06 to 0.25)	0.13 (−0.11 to 0.36)
Gestational age, wk									
Very and moderate preterm (<35)	67 214 (2.7)	10.32 (7.00)	70 351 (2.8)	10.38 (6.95)	76 994 (2.7)	10.42 (6.99)	0.10 (0.03 to 0.17)	0.08 (0.01 to 0.16)	−0.02 (−0.12 to 0.09)
Late preterm (35-36)	179 705 (7.3)	12.22 (7.00)	190 637 (7.7)	12.25 (6.98)	214 568 (7.5)	12.30 (7.02)	0.08 (0.03 to 0.12)	0.12 (0.07 to 0.16)	0.04 (−0.02 to 0.10)
Term (37-41)	2 105 914 (85.1)	13.45 (6.79)	2 100 087 (84.5)	13.43 (6.78)	2 410 281 (84.6)	13.47 (6.78)	0.03 (0.01 to 0.04)	0.08 (0.07 to 0.10)	0.06 (0.04 to 0.08)
Postterm (>41)	122 989 (5.0)	13.66 (7.17)	125 047 (5.0)	13.64 (7.11)	145 749 (5.1)	13.66 (7.12)	0.07 (0.01 to 0.12)	0.12 (0.06 to 0.17)	0.05 (−0.02 to 0.12)

Abbreviations: APNCU, adequacy of prenatal care utilization; BMI, body mass index (calculated as weight in kilograms divided by height in meters squared).

^a^ We mutually adjusted all variables in the table in linear regressions.

^b^ Net changes during the pandemic were calculated as GWG for 2020 vs 2019 minus GWG for 2019 vs 2018, and the corresponding 95% CIs were calculated as the square root of the sum of the squares of the separate SEs.

^c^ Includes non-Hispanic American Indian or Alaskan Native, non-Hispanic with more than 1 race, and unknown or undisclosed race or ethnicity.

^d^ The APNCU index was calculated based on the month in which prenatal care is initiated and the number of visits from initiation of care until delivery. It is categorized into 4 levels: *inadequate* care is defined as starting prenatal care after the fourth month of pregnancy or receiving less than 50% of expected visits based on the schedule of prenatal care visits recommended by the American College of Obstetricians and Gynecologists; *intermediate* care is care begun by month 4 with 50% to 79% of expected visits received; *adequate* care is begun by month 4 with 80% to 109% of expected visits received; and *adequate plus* care is begun by month 4 with 110% or more of expected visits received.

**Table 2.** Changes in Risk of Excessive Gestational Weight Gain Before and During the COVID-19 Pandemic by Maternal Characteristics^a^

Maternal characteristic	OR (95% CI)^b^		Ratio of OR (95% CI)^c^	*P* value for effect modification^d^
	2019 vs 2018	2020 vs 2019		
Total	1.01 (1.00-1.01)	1.02 (1.01-1.02)	1.01 (1.01-1.02)	NA
Age, y				
<25	1.01 (1.00-1.02)	1.06 (1.05-1.07)	1.05 (1.04-1.06)	[Reference]
25-29	1.01 (1.02-1.02)	1.02 (1.01-1.03)	1.01 (1.00-1.02)	<.001
30-34	1.00 (1.0-1.01)	1.00 (0.99-1.01)	1.00 (0.99-1.01)	<.001
≥35	1.00 (0.99-1.01)	0.99 (0.98-1.00)	0.99 (0.98-1.00)	<.001
Educational attainment				
High school or less	1.01 (1.00-1.02)	1.04 5(1.04-1.05)	1.03 (1.03-1.04)	[Reference]
Some college	1.01 (1.00-1.02)	1.05 (1.04-1.05)	1.03 (1.02-1.04)	.78
Associate’s degree	1.01 (0.10-1.02)	1.02 (1.01-1.04)	1.01 (1.00-1.03)	.04
Bachelor’s degree	1.00 (0.99-1.01)	0.98 (0.97-0.99)	0.98 (0.97-0.99)	<.001
Master’s degree or higher	1.00 (0.98-1.01)	0.95 (0.94-0.96)	0.96 (0.94-0.97)	<.001
Unknown	0.99 (0.96-1.02)	1.04 (1.01-1.08)	1.06 (1.01-1.11)	.38
Race or ethnicity				
Hispanic	1.01 (1.00-1.02)	1.03 (1.02-1.03)	1.02 (1.00-1.03)	.23
Non-Hispanic Black	1.01 (1.00-1.02)	1.04 (1.03-1.05)	1.03 (1.01-1.04)	.01
Non-Hispanic White	1.00 (1.00-1.01)	1.01 (1.00-1.01)	1.01 (1.00-1.01)	[Reference]
Other^e^	1.00 (0.98-1.02)	1.06 (1.04-1.08)	1.06 (1.03-1.08)	<.001
Marital status				
Married	1.00 (1.00-1.01)	1.00 (0.99-1.00)	0.99 (0.99-1.00)	[Reference]
Unmarried	1.01 (1.00-1.01)	1.04 (1.04-1.05)	1.04 (1.03-1.05)	<.001
Unknown	1.01 (1.00-1.02)	1.03 (1.02-1.04)	1.02 (1.00-1.03)	.008
APNCU index^f^				
Inadequate	1.01 (1.00-1.02)	1.03 (1.02-1.04)	1.02 (1.00-1.03)	[Reference]
Intermediate	1.00 (0.98-1.02)	1.04 (1.02-1.06)	1.04 (1.01-1.07)	.18
Adequate	1.00 (0.99-1.01)	1.03 (1.02-1.04)	1.03 (1.02-1.05)	.15
Adequate plus	1.01 (1.00-1.01)	1.01 (1.01-1.02)	1.01 (1.00-1.01)	.16
Unknown	1.03 (1.01-1.06)	0.99 (0.97-1.02)	0.96 (0.93-0.99)	.002
BMI				
Normal weight (18.5-24.9)	1.00 (1.00-1.01)	1.00 (0.99-1.00)	0.99 (0.98-1.00)	[Reference]
Underweight (<18.5)	1.04 (1.02-1.06)	1.00 (0.98-1.02)	0.96 (0.93-1.00)	.09
Overweight (25.0-29.9)	1.02 (1.00-1.02)	1.01 (1.00-1.02)	1.00 (0.99-1.01)	.09
Obesity (≥30)	1.01 (1.00-1.02)	1.05 (1.05-1.06)	1.04 (1.04-1.06)	<.001
Payment source for delivery				
Medicaid	1.01 (1.00-1.01)	1.05 (1.04-1.05)	1.04 (1.03-1.05)	[Reference]
Private insurance	1.01 (1.00-1.01)	0.99 (0.99-1.00)	0.99 (0.98-0.99)	<.001
Self-pay	1.02 (1.00-1.04)	1.02 (1.00-1.04)	1.00 (0.98-1.03)	.01
Other	1.01 (0.99-1.03)	1.04 (1.02-1.06)	1.03 (1.01-1.06)	.68
Unknown	0.99 (0.93-1.04)	1.00 (0.96-1.05)	1.02 (0.95-1.10)	.60
Gestational age				
Term (37-41 wk)	1.00 (1.00-1.01)	1.02 (1.01-1.02)	1.01 (1.00-1.02)	[Reference]
Very and moderate preterm (<35 wk)	1.02 (0.99-1.04)	1.02 (1.00-1.04)	1.00 (0.97-1.04)	.72
Late preterm (35-36 wk)	1.02 (1.00-1.03)	1.04 (1.02-1.05)	1.02 (1.00-1.04)	.28
Postterm (>41 wk)	1.01 (1.00-1.03)	1.02 (1.01-1.04)	1.01 (0.99-1.03)	>.99

Abbreviations: APNCU, adequacy of prenatal care utilization; BMI, body mass index (calculated as weight in kilograms divided by height in meters squared); NA, not applicable; OR, odds ratio.

^a^ Excessive gestational weight gain was defined as weight gain above the BMI-specific Institute of Medicine recommendations.

^b^ We mutually adjusted all covariates in the table in logistic regressions.

^c^ Log ORs were calculated as log gestational weight gain for 2020 vs 2019 minus log gestational weight gain for 2019 vs 2018, and the corresponding SEs were calculated as the square root of the sum of the squares of the separate logs of SEs.

^d^ *P* < .05 indicates statistically significant log OR (at 5% level) compared with the reference group.

^e^ Includes non-Hispanic American Indian or Alaskan Native, non-Hispanic with more than 1 race, and unknown or undisclosed race or ethnicity.

^f^ The APNCU index was calculated based on the month in which prenatal care is initiated and the number of visits from initiation of care until delivery. It is categorized into 4 levels: (1) *inadequate* care is defined as starting prenatal care after the fourth month of pregnancy or receiving less than 50% of expected visits based on the schedule of prenatal care visits recommended by American College of Obstetricians and Gynecologists; (2) *intermediate* care is care begun by month 4 with 50% to 79% of expected visits received; (3) *adequate* care is begun by month 4 with 80% to 109% of expected visits received; and (4) *adequate plus* care is begun by month 4 with 110% or more of expected visits received.

## Discussion

These findings suggest that the COVID-19 pandemic was associated with higher GWG and higher risk of excessive GWG among US individuals with singleton pregnancies, especially those younger than 25 years, non-Hispanic Black individuals, unmarried individuals, individuals with obesity before pregnancy, and individuals using Medicaid to pay for delivery. These findings shed light on the associations of the pandemic with adverse pregnancy outcomes^5^ and highlight the need to address pandemic-related GWG, particularly among vulnerable populations, to minimize the public health impact. Study limitations include self-reported height and weight before pregnancy and lack of information on COVID-19 infection on birth certificates. Future studies that identify the period of maximum association of the COVID-19 pandemic with GWG may be useful.

## Notes

Supplement. **eAppendix.** Statistical Analysis
eReferences
