# Supplementary material for: Analysis of Gestational Weight Gain During the COVID-19 Pandemic in the US
Source: JAMA Netw Open. 2022 Sep 9;5(9):e2230954. doi: 10.1001/jamanetworkopen.2022.30954 (PMC9463602; doi:10.1001/jamanetworkopen.2022.30954)
Supplement: Supplement. — eAppendix. Statistical Analysis eReferences [file jamanetwopen-e2230954-s001.pdf]

Supplementary Online Content

Cao W, Sun S, Danilack VA. Analysis of gestational weight gain during the COVID-19 pandemic in the US. *JAMA Netw Open*. 2022;5(9):e2230954. doi:10.1001/jamanetworkopen.2022.30954

eAppendix. Statistical Analysis
eReferences

This supplementary material has been provided by the authors to give readers additional information about their work.

© 2022 Cao W et al. *JAMA Network Open.*

eAppendix. Statistical Analysis

We used linear regression to estimate changes in GWG by comparing GWG in 2020 vs. 2019 (i.e., $GWG_{2020 \text{ vs. } 2019}$) and GWG in 2019 vs. 2018 (i.e., $GWG_{2019 \text{ vs. } 2018}$). We used logistic regression to estimate odds ratio (OR) of excessive GWG by comparing excessive GWG in 2020 vs. 2019 ($eGWG_{2020 \text{ vs. } 2019}$) and excessive GWG in 2019 vs. 2018 ($eGWG_{2019 \text{ vs. } 2018}$). All models were adjusted for gestational age (very and moderate preterm, late preterm, term, and postterm), maternal age (<25, 25-29, 30-34, and 35+ years), educational attainment (high school or less, some college, associate degree, bachelor's degree, master's degree or higher, and unknown), race/ethnicity (non-Hispanic white, non-Hispanic black, Hispanic, Asian or Pacific Islander, and other), marital status (married, unmarried, and unknown), adequacy of prenatal care utilization (APNCU) index (inadequate, intermediate, adequate, adequate plus, and unknown), pre-pregnancy body mass (normal weight, underweight, overweight, and obese), and source of delivery payment (Medicaid, private insurance, other, and unknown).

Gestational age was categorized into very and moderate preterm (gestational age less than 35 weeks); late preterm (gestational age of 35 weeks to 36 weeks); term (gestational age of 37 weeks to 41 weeks); and postterm (gestational age above 41 weeks). The APNCU index determines the adequacy of prenatal care utilization based on two parts: the month in which prenatal care is initiated and the number of visits from initiation of care until delivery. It is categorized into four levels: "Inadequate" care is defined as either starting prenatal care after the 4th month of pregnancy or receiving less than 50% of expected visits based on the schedule of prenatal care visits recommended by American College of Obstetricians and Gynecologists (ACOG). "Intermediate" care is care begun by month 4 and with 50–79% of expected visits received; "adequate" care is that begun by month 4 and with 80–109% of expected visits received; "adequate plus" care is begun by month 4 and with 110% or more of expected visits received.[1] Pre-pregnancy BMI was categorized into underweight (BMI less than 18.5); normal weight (BMI 18.5-24.9); overweight (BMI 25.0-29.9); and obese (BMI greater than or equal to 30.0).

We calculated GWG associated with the pandemic by excluding pre-pandemic trends in GWG. We calculated net change due to pandemic for GWG (continuous outcome) and ratio of OR for excessive GWG (dichotomous outcome).[2]

For net change due to pandemic:
Estimate= $GWG_{2020 \text{ vs. } 2019}$ - $GWG_{2019 \text{ vs. } 2018}$
The corresponding standard errors for $GWG_{2020 \text{ vs. } 2019}$ and $GWG_{2019 \text{ vs. } 2018}$ is SE($GWG_{2020 \text{ vs. } 2019}$) and SE($GWG_{2019 \text{ vs. } 2018}$), then the standard error for the estimate is SE(estimate)=sqrt(SE($GWG_{2020 \text{ vs. } 2019}$)^2+ SE($GWG_{2019 \text{ vs. } 2018}$)^2), which is the square root of the sum of the squares of the separate standard errors.

For log of ratio of odds ratio:
Estimate= log $eGWG_{2020 \text{ vs. } 2019}$ – log $eGWG_{2019 \text{ vs. } 2018}$
The corresponding standard errors for log $eGWG_{2020 \text{ vs. } 2019}$ and log $eGWG_{2019 \text{ vs. } 2018}$ is SE($eGWG_{2020 \text{ vs. } 2019}$) and SE($eGWG_{2019 \text{ vs. } 2018}$), then the standard error for the estimate is SE(estimate)=sqrt(SE($eGWG_{2020 \text{ vs. } 2019}$)^2+ SE($eGWG_{2019 \text{ vs. } 2018}$)^2), which is the square root of the sum of the squares of the separate standard errors.

For the subgroup analysis, we fitted separate linear regression or logistic regression to the data for each subgroup, and estimated subgroup-specific estimates of ratio of odds ratio. To test for statistically significant difference in subgroup estimates,[3] for example, in blacks vs. whites, we calculated:

$$Z = \frac{ROR_{black} - ORR_{white}}{\sqrt{se(ROR_{black})^2 + se(ORR_{white})^2}}$$

Where ROR represents ratio of odds ratio and se is the corresponding standard error.

© 2022 Cao W et al. *JAMA Network Open.*

eReferences

1. Kotelchuck M. An evaluation of the Kessner adequacy of prenatal care index and a proposed adequacy of prenatal care utilization index. *Am J Public Health.* 1994;84(9):1414-1420.
2. Altman DG, Bland JM. Interaction revisited: the difference between two estimates. *BMJ.* 2003;326(7382):219. doi:10.1136/bmj.326.7382.219
3. Di Q, Dai L, Wang Y, et al. Association of short-term exposure to air pollution with mortality in older adults. *Jama.* 2017;318(24):2446-2456.

© 2022 Cao W et al. *JAMA Network Open.*
